# Precise micro-particle and bubble manipulation by tunable ultrasonic bottle beams

**DOI:** 10.1016/j.ultsonch.2021.105602

**Published:** 2021-05-21

**Authors:** Qinxin Zhou, Meiying Li, Chiyuan Fu, Xuemei Ren, Zheng Xu, Xiaojun Liu

**Affiliations:** a Institute of Acoustics, Tongji University, Shanghai 200092, China; b Department of Ultrasound, Shanghai University of Traditional Chinese Medicine Affiliated Putuo Hospital, Shanghai 200062, China; c School of Electronic and Information Engineering, Tongji University, Shanghai 200092, China; d Key Laboratory of Modern Acoustics, School of Physics, Nanjing University, Nanjing 210093, China

**Keywords:** Bubble manipulation, Acoustic radiation force, Ultrasonic lens, Bottle beams

## Abstract

This paper reports a method to generate tunable bottle beams using an ultrasonic lens, by which the bottle position can be precisely adjusted with the change of the acoustic frequency. Therefore, the position of a single particle or bubble in liquid can be manipulated without using phased array which is costly and huge with complex circuits. Furthermore, we introduced this method to multiple bubble manipulation using acoustic holography. The bottle properties against frequency are theoretically and experimentally analyzed. It is shown that the bottle position depends almost linearly on the operating frequency, which provides a basis for the precise manipulation of bubbles and particles. In addition, the relationship between the acoustic radiation force and the drag force under different incident acoustic pressures is considered, establishing a limit on the moving velocity of the trapped particles. The ultrasonic field observation is further demonstrated by Schlieren imaging system. The proposed method has potential biomedical applications, such as more flexible cell manipulation and targeted drug delivery in vivo, as well as potential applications in the study of chemical reactions between micro objects.

## Highlights

• Manipulation of particles (bubbles) with acoustic radiation force (Bjerknes force).
• Implementation of multi particle manipulation.
• Study of the change of particle position with operating frequency.
• Visualization of acoustic field by Schlieren imaging method.

## 1 Introduction

There is a need to manipulate micrometer-sized particles or cells in many areas of physics, analytical chemistry, and biological sciences [1]. In contrast with optical [2], [3], [4], electrical [5], [6], [7] or magnetic methods [8], [9], acoustic methods for particle manipulation are ideal for biomedical applications. Because sound is a mechanical wave, it can be transmitted easily into biological tissue with low attenuation and high precision. Other advantages of acoustic particle manipulation include its high safety for use with biological objects (such as cells), no requirement for pretreatment of particles, and its ability to manipulate almost any type of microscale particles regardless of their electrical or magnetic properties. Thus, acoustic methods are widely considered to be promising for manipulating cells and drug delivery in vivo [10]. In addition, ultrasonic stimulation is always accompanied by the generation of cavitation bubbles [11], [12], [13], [14], [15]. The manipulation of single and multiple bubbles is conducive to the study of sonochemical reaction [16], [17], [18].

The fundamental theory of acoustic waves acting on particles was developed by King in 1934 [19]. More recently, researchers have proposed many methods to manipulate particles [20], [21], [22], [23], [24], [25], [26], [27], [28], [29], and the precise manipulation of single particles and cells has become increasing important in life science and medicine. To this end, research on particle manipulation by surface acoustic wave devices has been carried out [30], [31], [32]. However, these methods are difficult to be used in vivo. Phased-array transducers control the excitation of multiple independent transducer elements to shape acoustic fields to accurately manipulate single particles in three dimensions [33], [34], [35], [36], [37], [38], [39]. However, their circuits are complex and the array elements occupy a large space. Thus, researchers proposed the use of acoustic holograms to trap multiple particles by shaping acoustic waves transmitted through a structured phase plate [40], [41].

There is also a need to contactless manipulate individual microbubbles, which are important in sonochemical reaction and sonoluminescence. Excluding buoyancy, the most potent driving forces for bubble translations in a liquid is the Bjerknes forces. It generates from strong periodic change in volume of bubbles due to external pressure oscillations and controls the bubble translational motion. Theoretically, Leighton [42] conducted the calculation for primary Bjerknes forces in the standing wave field. The results indicate that bubbles larger or smaller than the resonance radius move to the nodes or antinodes of the standing wave, respectively. Experimentally, Ashokkumar [43], [44], [45], [46] and co-authors systematically investigated the motion of bubbles in the acoustic field. Meng et al. realized the manipulation of microbubbles by two-dimensional surface standing waves [47]. One step more, Baresch and Garbin [34], [48] trapped and manipulated a single microbubble in 3D with eight independent transducers driven with the necessary time delay to construct a single-beam acoustical tweezer. However, the balance of the bubble in vertical axis is the balance between Bjerknes force and buoyancy force, which causes the bubble in different size will be trapped in different places and the position of the bubble becomes difficult to be adjusted by the change of the ultrasonic frequency.

In this work, we propose a tunable bottle-beam ultrasonic lens. Using this lens, the position of the bottle can be controlled axially by adjusting the operating frequency to realize the accurate movement of a single particle or microbubble. The paper is organized as follows: In section 2.1, the design principle of bottle beam ultrasonic lens is introduced. The forces exerted on the particles or bubbles by the generated bottle beam are shown and discussed in 2.2, 2.3. The simulation and experimental methods of ultrasonic field, flow field and particle/bubble trajectory are presented in 2.4, 2.5, respectively. Simulation results of ultrasonic field, flow field and particle field, as well as experimental results of ultrasonic field and particle field are presented and discussed in section 3. The paper is finalized with our conclusions in section 4.

## 2 Theory and methods

### 2.1 Generation of bottle beams

**Fig. 1:**
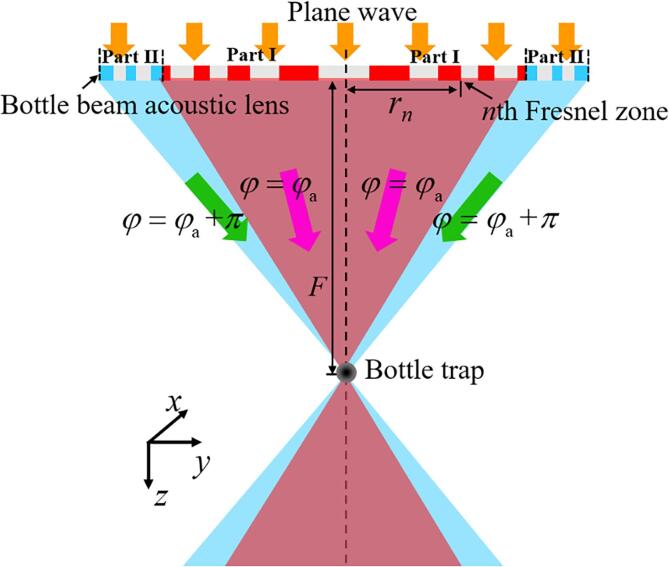
Schematic diagram showing wave propagation through the bottle-beam lens.

Fig. 1 shows a schematic diagram of the ultrasonic lens for achieving a single bottle beam. The material of the lens was photosensitive resin. When the ultrasonic waves emitted by the transducer plate (impedance $Z_{\text{t}}=\rho_{\text{t}}c_{\text{t}}$, $\rho_{\text{t}}$= 1600 kg/m^3^, $c_{\text{t}}$= 2305 m/s) pass through the photosensitive resin ($\rho_{\text{r}}$=1160 kg/m^3^, $c_{\text{r}}$=2250 m/s) to the water ($\rho_{\text{w}}$=998 kg/m^3^, $c_{\text{w}}$= 1482 m/s), the intensity transmission coefficient can be expressed as

(1) $$T_{I}=\frac{4Z_{\text{t}}Z_{w}}{{\left(Z_{\text{t}}+Z_{\text{w}}\right)}^{2}\cos^{2}\left(k_{\text{r}}D\right)+{\left(Z_{\text{r}}+\frac{Z_{\text{t}}Z_{\text{w}}}{Z_{\text{r}}}\right)}^{2}\sin^{2}k_{\text{r}}D},$$

where $k_{\text{r}}\;\left(=2\pi /\lambda_{\text{r}}\right)$ is the wave number of the photosensitive resin, and $\lambda_{\text{r}}$and *D* are its wavelength and thickness, respectively. For the 2.2-mm thick photosensitive resin used, the phase of the original acoustic pressure can be corrected and contributes to phase cancellation at the target point. We calculated a transmittance of 0.94 using Eq. (1). Therefore, there was strong ultrasonic intensity gain at the target.

The phase difference ($\varphi_{n}\left(z\right)$) between the ultrasonic waves transmitted from the center and the *n*th Fresnel zone can be expressed as [49]:

(2) $$\varphi_{n}\left(z\right)=k_{\text{w}}\cdot \left(\sqrt{z^{2}+r_{n}^{2}}-z\right),$$

where $r_{n}$ (*n* = 1, 2, 3, …) is the radius of each zone. To generate a bottle beam, the lens was divided into two parts, as shown in Fig. 1 (part Ⅰ and part Ⅱ**)**. The ultrasonic waves transmitted from part I and part II should be in the reverse phase when they reach the bottle center, which can be expressed as:

(3) $$\varphi_{n}\left(z\right)=\left\{\begin{array}{cc} 2n\pi & (r⩽r_{\text{s}}\text{)}\\ 2n\pi \;\text{+}\;\pi & \left(r>r_{\text{s}}\right) \end{array}\right),$$

where *r*_s_ is the energy-sharing point. Therefore, the ring radii can be calculated as:

(4) $$r_{n}=\left\{\begin{array}{cc} \sqrt{2n\lambda_{\text{w}}F+n^{2}\lambda_{\text{w}}^{2}} & \left(r⩽r_{\text{s}}\right)\\ \sqrt{n\lambda_{w}F+\frac{n^{2}}{4}\lambda_{w}^{2}} & \left(r>r_{\text{s}}\right) \end{array}\right),$$

where *F* is the bottle depth.

### 2.2 Acoustic forces exerted on particles

The generated bottle beam exerts forces on the particles to trap and manipulate them. Particles in the acoustic field are subjected to two acoustic forces: the Stokes drag force from the induced acoustic streaming flow, and the acoustic radiation force from the scattering of acoustic waves from the particles [20]. The time-averaged Stokes drag force,${\vec{F}}_{\text{drag}}$, on a spherical object of radius *a* moving with velocity ${\vec{u}}_{\text{p}}$ in a fluid with streaming velocity ${\vec{u}}_{\text{w}}$ is given by:

(5) $${\vec{F}}_{\mathrm{drag}}=6\pi \eta a\left({\vec{u}}_{w}-{\vec{u}}_{p}\right),$$

where η is the viscous coefficient of the fluid. The time-averaged acoustic radiation force,${\vec{F}}_{\text{rad}}$, on the same small spherical particle is given by [50]:

(6) F→rad=-πa3[2κw3ReS0∗pa∗∇pa-ρwReS1∗u→o∗∇u→o],

where *κ*_w_ denotes the isentropic compressibility of the fluid; *S*_0_ and *S*_1_ denote the monopole and dipole scattering coefficients of suspended particles, respectively; the asterisk denotes complex conjugation; and *p*_a_ and ${\vec{u}}_{\text{o}}$ are the incident acoustic pressure and oscillation velocity at the particle position, respectively. The nominal radius, density, speed of sound, Poisson’s ratio, and compressibility of the polystyrene particles were 150 μm, 1050 kg/m^3^, 2350 m/s, 0.35, and 249 T/Pa, respectively.

Because the magnitude and direction of the acoustic radiation force on particles are determined by their acoustic contrast factor (related to their density and compressibility), single-particle trapping can take two forms. For rigid particles, if the speed of sound through them is much higher than that through the medium, the direction of the radiation force points to the maximum (minimum) acoustic intensity when *ρ*_p_ < 0.4*ρ*_m_ (*ρ*_p_ > 0.4*ρ*_w_), and the particles are bound in the focus (bottle center) [20].

### 2.3 Bjerknes force exerted on bubbles

When a gas bubble in liquid is subjected to an acoustic pressure field, it can undergo radius pulsations (*R*(*t*)) described by the RPNNP equation:

(7) $$R\ddot{R}+\frac{3}{2}{\dot{R}}^{2}=\frac{1}{\rho_{\text{w}}}\left[\left(P_{0}+\frac{2\sigma }{R_{0}}-P_{\text{V}}\right){\left(\frac{R_{0}}{R}\right)}^{3\kappa }-\frac{2\sigma }{R}-\frac{4\eta \dot{R}}{R}-P_{0}-P\left(t\right)\right],$$

where *R*_0_ is the equilibrium bubble radius, *P*_0_ (=1.013 × 10^5^ Pa) is the hydrostatic pressure, *P*_v_ is the vapour pressure, and σis the liquid surface tension. The polytropic index of the gas within the bubble is given by κ, and *P*(*t*) is the time-varying acoustic pressure. Thus, if the acoustic pressure gradient (∇P(z,t)) is non-zero, and then it can couple with the oscillation of the bubble volume (*V*(*t*)) to produce a translational force on the bubble. This is the primary Bjerknes force, which is written as [11], [42]:

(8) $$F_{\text{b}}=-V\left(t\right)\nabla P\left(z,t\right)$$

According to the conclusion conducted by Leighton, bubbles move to the intensity maximum or minimum depends on their radius smaller or greater than the resonance radius (*b*). It is calculated using the Minnaert equation [51]:

(9) $$b=\frac{1}{2\pi f_{\text{op}}}{\left(\frac{3\chi P_{0}}{\rho_{w}}\right)}^{\frac{1}{2}},$$

where *f*_op_ is the operating frequency, andχ (=1.4) is the polytropic index of the gas within the bubble. For an acoustic frequency of 1 MHz, the resonance radius (*b*) is about 3.3 μm. Here, we are focused on the trap of bubbles of radius *R*_b_ > *b* which will move to the minimum acoustic intensity induced by a bottle beam.

### 2.4 Numerical simulations

To verify that the designed ultrasonic lens can be used to trap single particles, we present an idealized numerical model, and describe how we achieved the coupling calculation of the ultrasonic field, radiation force field, flow field, and particle trajectory using the finite-element software COMSOL Multiphysics. Calculations were carried out in a rectangular tank filled with water. To reduce the burden of the simulation, an axis-symmetric definition of the geometry was used in the simulation of the ultrasonic field and flow field. The transducer was mounted on the upper central part of the tank. The transducer plate was set as the incident radiation pressure. An absorber was placed at the bottom of the tank, which was set as the perfect matched layer. We input the volume force caused by the acoustic radiation force acting on the fluid elements as the domain condition and non-slip as the boundary condition of each wall for the calculation of the flow field. The buoyancy of the particle is neglected since the particle used in the experiment has the similar density to the water. The time-dependent change of the particle position was determined using the COMSOL particle-tracing module, only taking into account the drag force (Eq. (5)) and radiation force (Eq. (6)). The release position of single particles was the position of the bottle center at the given frequency. The boundary condition of the transducer, lens surface, and tank walls in the particle-tracing module was the bounce wall.

### 2.5 Experimental details

**Fig. 2:**
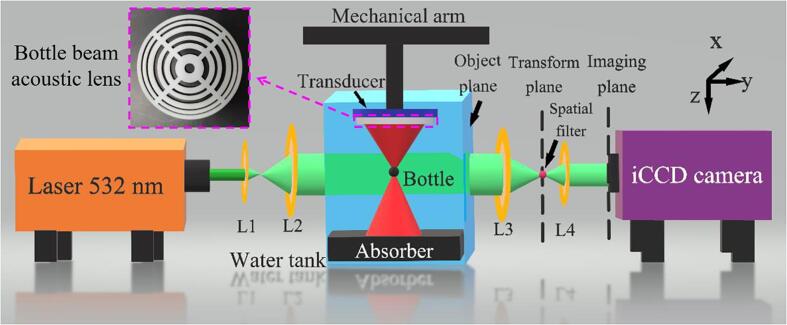
Schematic illustration of the experimental setup used for Schlieren imaging.

To confirm the simulation results of the ultrasound field, we used the Schlieren imaging method to visualize it [52], [53], [54], [55], [56], [57]. This method is based on the phase modulation of light produced by the acousto-optic effect. A schematic diagram of the experimental setup used for Schlieren imaging is shown in Fig. 2. A laser beam (532 nm, DPGL-2150F, Photop, China) was expanded as a collimated light beam with a 75-mm diameter using lenses L1 and L2, which were incident to the ultrasound field. The ultrasound field was generated in a transparent rectangular water tank made of quartz glass with dimensions of 100 × 200 × 200 mm^3^ (width × length × height). The rear surface of the tank was placed at the front focal plane of lens L3 and served as the object plane, and the upper surface of the water tank was open. To visualize the ultrasonic field, a circular plate, which blocked zero-order diffraction light, was placed on the transform plane. An intensified charge-coupled device (iCCD) camera (iStar DH734-18U-03, Andor, UK) was placed on the imaging plane to record the images.

A planar wave field was generated using an ultrasonic transducer (DYG-1 M, Dayu Electric, China) with a radius and a center frequency of 25 mm and 1 MHz, respectively. The bottle-beam ultrasonic lens was fixed to the transducer surface and immersed in water (distilled water without degassing). The lens used in the experiment is shown in the purple dotted frame in Fig. 2. To create travelling waves, the polyurethane wedge absorber was placed at the bottom of the water tank. The distance between the transducer and the absorber was 95 mm. The transducer was driven by a signal generator (AFG3021, Tektronix, USA) and a power amplifier (ATA-122D, Aigtek, China). The incident pressure amplitude radiated from the transducer was measured to be approximately 0.9 × 10^5^ Pa.

## 3 Results and discussion

**Fig. 3:**
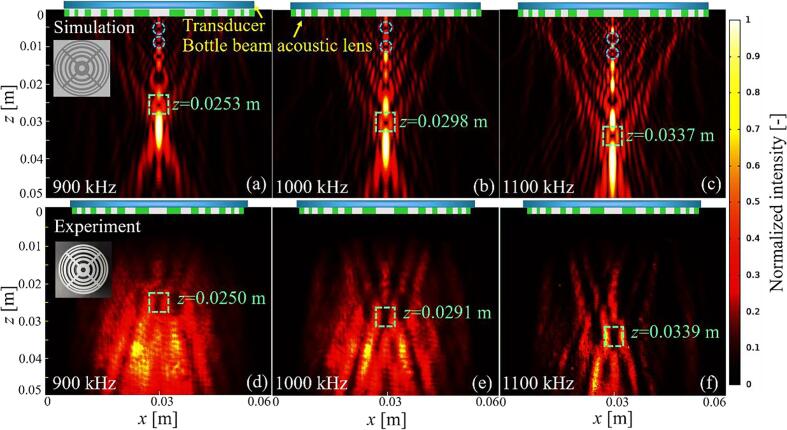
(a–c) Simulation and (d–f) experimental results for the intensity distribution of the acoustic field at frequencies of 900, 1000, and 1100 kHz. The bottle changes with different operating frequencies in the axial direction can be observed.

Fig. 3 shows the simulated and experimental intensity distribution of the ultrasonic field generated by the lens on the *x*–*z* plane. When the ultrasonic waves are emitted from the lens (the 3D model and photograph of the lens are shown in the upper left corner of Fig. 3 **(a)** and **(d)**, respectively), a bottle (shown in the green dotted frames) surrounded by a high-intensity ultrasonic field appears in all directions. This figure also shows that the position of the bottle will change accordingly with the change of the operating frequency. According to Eq. (4), the position of the bottle at the operating frequency can be obtained by

(10) $$F\;\text{=}\;\frac{R^{2}}{2nc_{\text{w}}}f_{\text{op}}-\frac{nc_{\text{w}}}{2f_{\text{op}}},$$

where *R* is the external radius of the bottle-beam ultrasonic lens, and *c*_w_ is the speed of sound in water. The theoretical bottle position corresponding to the three frequencies of 900, 1000, and 1100 kHz considered in Fig. 3 are 0.0267, 0.0307, and 0.0346 m, respectively, which agree well with the experimental results.

A special feature of the lens is that it has multiple bottles at F/m, where *m* is an odd integer (as shown in the blue dotted frames in Fig. 3 **(a–c)**). When the *z*-coordinate moves from the main bottle to the ultrasonic lens, each open zone is re-divided into *m* half-wave zones. Only when *m* is an odd number, the destructive interference will occur between two adjacent half-wave zones with opposite phases, although there is still one remaining half-wave zone that contributes to the amplitude at the observation point. The two remaining half-wave zones with opposite phases in part I and part II interfere destructively to form a bottle. The main bottle at one frequency may coincide with the secondary bottle at another frequency. However, with increasing number of half-wave zones in each open zone, the effective half-wave zone area decreases, which weakens the acoustic intensity gradient of the bottle. Therefore, the acoustic intensity gradient in the primary bottle is the largest, which makes it easier for the particles to be trapped stably.

**Fig. 4:**
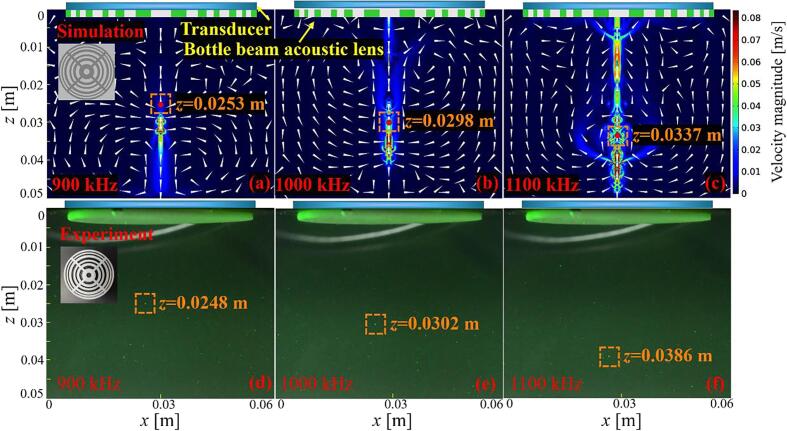
(a–c) Simulation results of the flow field and particle trajectory, and (d–f) experimental results of the particle trajectory at frequencies of 900, 1000, and 1100 kHz.

After obtaining the ultrasonic field, the flow field and particle trajectory (Fig. 4 **(a–c)**) were simulated, and the particle/bubble manipulation experiment (Fig. 4 **(d–f**) was conducted. Because the density of the particle we used satisfies the condition of *ρ*_p_ > 0.4*ρ*_w_, it will be pushed to the position of minimum acoustic intensity (i.e., it can be stably trapped in the intensity null surrounded by the high-intensity ultrasonic field in all directions) in the bottle beam. Both simulated and experimental results show that a single particle can be trapped in bottles corresponding to the frequencies in Fig. 3. It can be seen from Fig. 4 that when the operating frequency changes, the particle position changes accordingly (See also Supplementary Movie 1 (SM1), in which the ultrasonic field and particle trajectory vary with the operating frequency). In addition, SM1 also shows the bubble position varies with the operating frequency. Because the radius of the bubble here is 160 μm, which is much larger than that of the resonance radius *b,* the direction of the Bjerknes force points to the minimum ultrasonic intensity. Therefore, the bubble can be trapped in the bottle center and move as the operating frequency changed. We note that in SM1, although the bubble motion is more difficult to be controlled, it still moves according to the expected trajectory on the whole. The fluctuation of bubble motion is because it is more affected by buoyancy than solid particle.

**Fig. 5:**
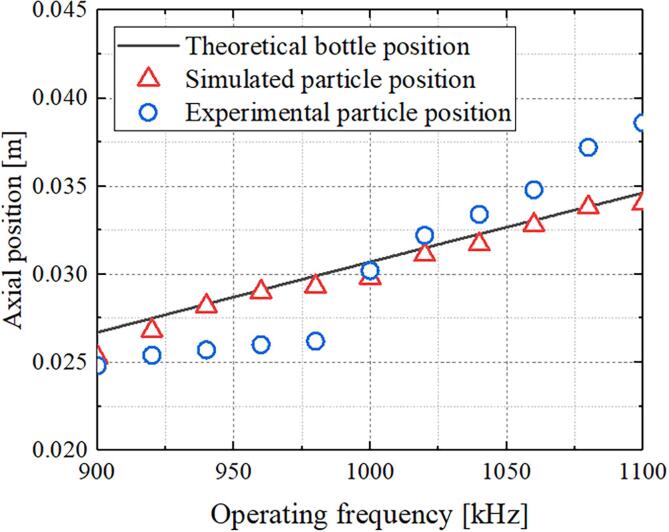
Axial position as a function of operating frequency.

We have calculated the bottle position as a function of the working frequency to better understand this relationship. From the dark solid line in Fig. 5, we conclude that there is a near linear dependence of the bottle position on the operation frequency. This is because the second term in Eq. (10) is close to zero for the range of operating frequencies considered. Thus, the result becomes more linear when either the frequency or the external radius is augmented or when the number of Fresnel zones is decreased. In Fig. 5, the red triangles correspond to the simulation results for operating frequencies from 900 to 1100 kHz with 20-kHz steps, and the blue circles represent the experimental results. The simulation results are in good agreement with the theoretical results. However, for the experimental results, although the particle position is consistent with the prediction at 1000 kHz, there is deviation at other frequencies. This is because when the operating frequency deviates from the center frequency (1000 kHz), the ultrasonic power radiated by the transducer decreases, which makes it not completely in the center of the bottle under the action of drag force, gravity and buoyancy.

To trap a particle with a density close to water or a bubble stably in the ultrasonic field, the acoustic radiation force on the particles or the Bjerknes force on the bubbles must satisfy certain conditions respectively. As for the particle trapping, the acoustic radiation force must be greater than the drag force, and thus the maximum manipulation velocity of the trapped particles can be given by

(11) $$u_{\max\text{p}}=\frac{F_{\text{rad}}}{6\pi \eta a}-u_{\text{w}}.$$

When the trapped particles move at a velocity lower than this maximum, they can be manipulated. Otherwise, they may escape from the bottle because the acoustic radiation force is less than the drag force.

**Fig. 6:**
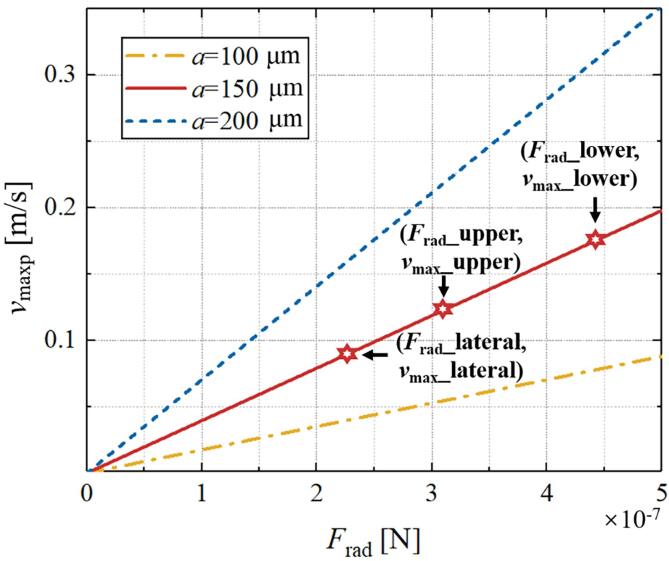
Values of maximum velocities for the considered *F*_rad_-*a* value pairs.

According to Eq. (11), we can calculate the maximum velocity *v*_maxp_ (=*u*_maxp_ + *u*_w_) for the considered *F*_rad_-*a* value pairs, as shown in Fig. 6. It should be noted that the value of the radiation force (*F*_rad_) is related to the particle radius (*a*), hence the change of *F*_rad_ should also be considered when calculating the relationship between *v*_maxp_ and *a*. In the previous simulation results (Fig. 4), we found that the ultrasonic intensity as well as radiation force in each direction around the bottle is different. Therefore, we selected three points with the largest radiation force in the simulation results, which are located on the lateral side, the upper side and the lower side of the bottle respectively (Since the left and right sides are symmetrical, we use ‘lateral side’ here). Their *F*_rad_ values are the abscissa values corresponding to the red hexagon stars in Fig. 6, and the *v*_maxp_ values are their ordinate values. For simplicity, the flow field in each direction can be approximately equal (*u*_w_ = 0.04 m/s). It can be seen from Fig. 6 that the radiation force (*F*_rad_) and the maximum velocity (*v*_maxp_) in the three directions around the bottle are both lateral side < upper side < lower side. However, when the particle moves up (down), the acoustic radiation force it receives comes from the lower side (upper side) of the bottle, thus the maximum moving speed of the particle (*u*_maxp_) is: transverse (0.0518 m/s) < downward (0.0871 m/s) < upward (0.1400 m/s).

Besides Bjerknes force and drag force, buoyancy force also affects the motion of bubbles. Therefore, the study of bubble movement is relatively more complex and should be divided into the following situations. When the bubble is moved laterally, the Bjerknes force must be greater than the drag force, thus the maximum velocity of the trapped bubble can be given by

(12) $$u_{\max\text{b}}=\frac{F_{\text{b}}}{6\pi \eta R_{\text{b}}}-u_{\text{w}}.$$

When the bubble is moved upward (downward), the upward buoyancy force (*F*_buo_) should also be considered, thus the maximum velocity of the trapped bubble can be given by

(13) $$u_{\max\text{b}}=\frac{F_{\text{b}}\pm F_{\text{buo}}}{6\pi \eta R_{\text{b}}}-u_{\text{w}}.$$

When the bubble is moved upward, the sum of Bjerknes force and buoyancy force must be greater than drag force, thus the sign before buoyancy should be positive, otherwise it is negative.

**Fig. 7:**
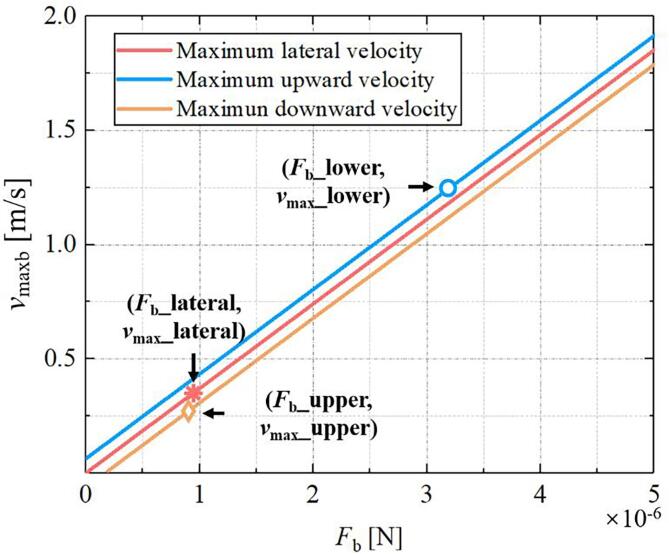
The relationship between the maximum velocity of bubble and the Bjerknes force (in three directions).

According to Eq. (12), (13), we can calculate the relationship between the maximum manipulation velocity *v*_maxb_ (=*u*_maxb_ + *u*_w_) and Bjerknes force (*F*_b_), as shown in Fig. 7. Here, the translation of the three oblique lines is due to buoyancy. It should be noted that as the particle moves, when the bubble moves upward (downward), the Bjerknes force it receives comes from the lower side (upper side) of the bottle. In the simulation results in Fig. 4, we selected the points with the maximum Bjerknes force on the lateral, upper and lower sides of the bottle. And their values are calculated with Eq. (8), which correspond to the abscissa values of red meter shaped, yellow diamond shaped and blue circle in Fig. 7. Finally, the maximum moving velocity (*u*_maxb_) of the bubble can be calculated as: downward (0.2623 m/s) < lateral (0.3119 m/s) < upward (0.8460 m/s) which indicates the bubble is trapped more tightly than the particle.

**Fig. 8:**
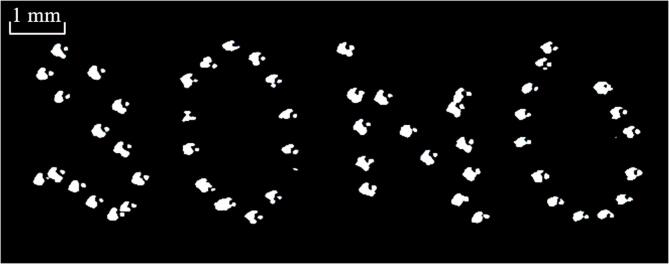
Bubble trajectory (‘SONO’, the acronym for *Ultrasonics Sonochemistry*) extracted from the experimental results.

According to the above results, we can change the position of the bottle by moving the transducer and the lens together using a mechanical arm, the velocity of which is less than the maximum manipulation velocity. This allows us to move the trapped particle or bubble in three dimensions. Fig. 8 shows the bubble moving trajectory extracted from the experimental results. The bubble was manipulated to move along the trajectory of the acronym for *Ultrasonics Sonochemistry* (SONO) (See Supplementary Movie 1).

**Fig. 9:**
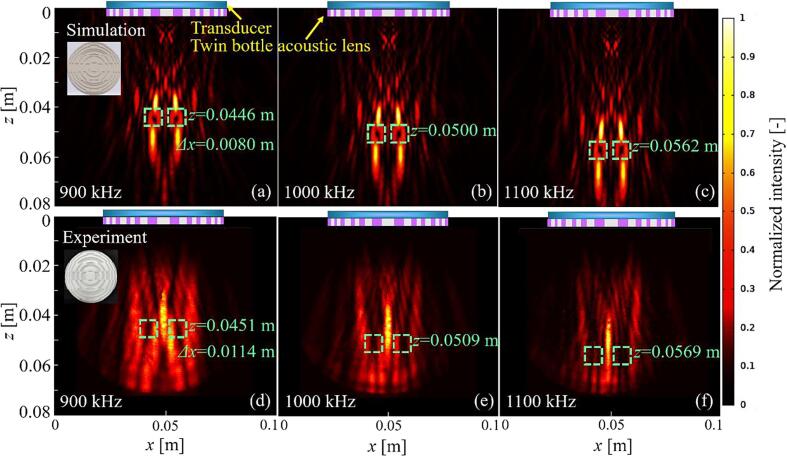
(a–c) Simulation and (d–f) experimental results for the intensity distribution of the twin bottle-beam field at frequencies of 900, 1000, and 1100 kHz.

By extending the concept to holographic technology, we obtained a twin bottle ultrasonic lens by inverting the target ultrasonic field. In general, the phase generated by the holographic lens changes continuously. We further set the phase when it was less than π as 0, and as π when it fell between π and 2π, and thus both sides of the structure were flat. In addition, because of the discontinuity of the lens structure, it is difficult to make it a lens by adding a connecting part directly. Therefore, we added a substrate thickness (1 mm) as phase 0, which also increased the thickness of phase π by 1 mm accordingly. The 3D-printing model and photograph of the lens are shown in the upper left corner of Fig. 9 **(a)** and **(d)**, respectively. The center and outer parts of the lens form two focusing points. The positions of the focusing points in these two parts are the same, but the phase between them is opposite. Hence, the focusing points are eliminated by the external focusing points owing to destructive interference. Therefore, when the ultrasonic waves pass through this lens, the reconstructed ultrasonic field is represented as a twin bottle beam.

Fig. 9 shows simulated and experimental intensity distribution of the twin bottle beam field on the *x–z* plane, with two bottles in each figure (shown in the green dotted frame). The longitudinal position of the two bottles can be simultaneously changed by adjusting the operating frequency of the transducer. It can be seen from the figure that the experimental results of the transverse position of the bottles and the distance between them are in good agreement with the simulation. Therefore, two particles or two bubbles can be trapped and manipulated simultaneously using this method.

Theoretically, arbitrary multiple bottle traps can be generated by the ultrasonic lens. However, with increasing number of bottles, the ultrasound intensity in each bottle decreases, thereby weakening the trapping force. There are three possible ways to steadily trap particles or bubbles while increasing the number of bottles. The first is to increase the incident acoustic power per unit area, which can enhance the ultrasound intensity of the target field and further increase the ultrasound intensity gradient to make the radiation force sufficiently large. The second method is to increase the radius of the incident field to increase the acoustic intensity converging to the bottle, although this is limited by the radius of the transducer. The third method is to increase the frequency of the incident wave to reduce the wavelength, such that the size of the bottle is also reduced, and hence increasing the gradient and further increasing the acoustic radiation force. However, for higher frequencies, the performance is limited by acoustic attenuation and the resolution of the 3D-printing process.

## 4 Conclusions

We have introduced a specific ultrasonic lens to generate position-adjustable bottle beams. In contrast with traditional bottle-beam lenses, ours is flat on both sides and can thus be perfectly attached to certain biological surfaces to ensure most of the ultrasonic waves transmit into tissue. Moreover, the structure can be described directly by a simple equation. The ultrasonic fields shaped using the proposed system allow us to simply and cost-effectively manipulate particles or bubbles without touching or contaminating them. As a supplement of using two-dimensional translation to realize the movement of particles and bubbles along the surface, the proposed bottle-beam can be moved along the central axis by adjusting the operating frequency. Therefore, it is suitable for the three-dimensional manipulation of cells or the axial transportation of drugs in vivo. In addition, this system allows multi-objects to be fixed simultaneously, which would enable the study of chemical interactions.

## Declaration of Competing Interest

The authors declare that they have no known competing financial interests or personal relationships that could have appeared to influence the work reported in this paper.
